# A survey of ticks (Acari: Ixodidae) of companion animals in Australia

**DOI:** 10.1186/s13071-016-1480-y

**Published:** 2016-05-10

**Authors:** Telleasha L. Greay, Charlotte L. Oskam, Alexander W. Gofton, Robert L. Rees, Una M. Ryan, Peter J. Irwin

**Affiliations:** Vector and Water-Borne Pathogen Research Laboratory, School of Veterinary and Life Sciences, Murdoch University, Perth, Western Australia Australia; Bayer Animal Health, Brisbane, Queensland Australia

**Keywords:** Ticks, Companion animals, Dogs, Cats, Horses, Tick-borne diseases, Australia

## Abstract

**Background:**

Ticks are among the most important vectors of pathogens affecting companion animals, and also cause health problems such as tick paralysis, anaemia, dermatitis, and secondary infections. Twenty ixodid species have previously been recorded on dogs, cats, and horses in Australia, including *Rhipicephalus sanguineus*, *Ixodes holocyclus* and *Haemaphysalis longicornis*, which transmit tick-borne diseases. A survey of hard ticks (Acari: Ixodidae) was conducted during 2012–2015 to investigate tick species that infest dogs, cats, and horses in Australia.

**Methods:**

Individual tick specimens were collected from dogs, cats and horses across Australia and sample collection locations were mapped using QGIS software. Ticks were morphologically examined to determine species, instar and sex. The companion animal owners responded to questionnaires and data collected were summarised with SPSS software.

**Results:**

A total of 4765 individual ticks were identified in this study from 7/8 states and territories in Australia. Overall, 220 larvae, 805 nymphs, 1404 males, and 2336 females of 11 tick species were identified from 837 companion animal hosts. One novel host record was obtained during this study for *Ixodes myrmecobii*, which was found on *Felis catus* (domestic cat) in the town of Esperance, Western Australia. The most common tick species identified included *R. sanguineus* on dogs (73 %), *I. holocyclus* on cats (81 %) and *H. longicornis* on horses (60 %).

**Conclusions:**

This study is the first of its kind to be conducted in Australia and our results contribute to the understanding of the species and distribution of ticks that parasitise dogs, cats, and horses in Australia. Records of *R. sanguineus* outside of the recorded distribution range emphasise the need for a systematic study of the habitat range of this species. Several incomplete descriptions of ixodid species encountered in this study hindered morphological identification.

**Electronic supplementary material:**

The online version of this article (doi:10.1186/s13071-016-1480-y) contains supplementary material, which is available to authorized users.

## Background

As haematophagous obligatory parasites of reptiles, birds, and mammals, ticks are among the most important vectors of pathogens affecting livestock, companion animals, and humans worldwide [1, 2]. Ticks transmit viruses, bacteria, and protozoa during blood feeding, which can compromise the health of the vertebrate host [3]. A variety of factors influence the susceptibility of companion animals to TBD, including exposure to questing ticks, the pet’s lifestyle, and ectoparasite control [4]. Some TBD of companion animals are zoonotic [5, 6], which in some circumstances may also place human owners at risk of infection. Furthermore, companion animals can act as sentinels for emerging TBD [7–9]. In 2013, it was estimated that there are a total of 4.2 million pet dogs, and 3.3 million pet cats in Australia [10].

Of the 896 recognised tick species worldwide [11] there are 70 species endemic to Australia: 14 soft tick (family Argasidae) and 56 hard tick (family Ixodidae) species [12]. While the majority of these ticks are unique to Australia, there are five species that have been introduced since European colonisation in the last 250 years with poultry (e.g. *Argas persicus*), horses (e.g. *Otobius megnini*), cattle (e.g. *Haemaphysalis longicornis* and *Rhipicephalus australis*), and dogs (e.g. *Rhipicephalus sanguineus*) [12]; however, *R. sanguineus* may have been introduced earlier than this [13].

To date, 20 ixodid species have been recorded on dogs, cats and horses in Australia (Table 1). Dogs are the primary hosts of *R. sanguineus*; however, native ticks such as *Ixodes cornuatus*, *Ixodes holocyclus*, and *Ixodes tasmani* are known to parasitise domestic dogs, as well as cats. Ixodids that usually feed on cattle (*H. longicornis* and *R. australis*) also feed on horses but, as with dogs and cats, horses can also be parasitised by native Australian ticks [14].

**Table 1:** Ticks (Acari: Ixodidae) previously recorded on dogs (*Canis lupus familiaris*), cats (*Felis catus*), and horses (*Equus ferus caballus*) in Australia

	Host species			
Tick species	*Canis lupus familiaris*	*Felis catus*	*Equus ferus caballus*	References
*Amblyomma moreliae*	✗	✗	✓	[46]
*Amblyomma triguttatum ornatissimum*	✗	✗	✓	[47]
*Amblyomma triguttatum queenslandense*	✓	✗	✗	[47]
*Amblyomma triguttatum triguttatum*	✓	✓	✓	[47–49]
*Bothriocroton auruginans*	✓	✗	✗	[46]
*Bothriocroton fimbriatum*	✗	✗	✓	[46]
*Bothriocroton hydrosauri*	✗	✗	✓^a^	[14]
*Haemaphysalis bancrofti*	✓	✓	✓	[50, 51]
*Haemaphysalis bremneri*	✗	✗	✓	[50]
*Haemaphysalis longicornis* ^b^	✓	✓	✓	[14]
*Haemaphysalis novaeguineae*	✗	✗	✓	[50]
*Ixodes australiensis*	✓	✗	✗	[52]
*Ixodes cornuatus*	✓	✓	✗	[52]
*Ixodes fecialis*	✗	✓	✗	[52]
*Ixodes hirsti*	✗	✓	✗	[52]
*Ixodes holocyclus*	✓	✓	✓	[52]
*Ixodes myrmecobii*	✓	✗	✓	[14]
*Ixodes tasmani*	✓	✓	✓	[52, 53]
*Rhipicephalus australis* ^b^	✓	✓	✓	[45, 54]
*Rhipicephalus sanguineus* ^b^	✓	✓	✗	[54]

^a^May be attributable to *Bothriocroton tachyglossi* [40, 88]

^b^Introduced species. All other species are native to Australia

As a result of its geographical isolation and robust biosecurity regulations, Australia is considered free of many of the TBD endemic to countries overseas. There are currently two TBD of dogs recognised in Australia; canine infectious cyclic thrombocytopenia (CICT) and canine babesiosis. *Anaplasma platys* is the causative agent of CICT and was detected in dogs in central Australia in the early 2000s [15, 16]. Canine babesiosis is caused by *Babesia vogeli* and *Babesia gibsoni* in Australia. *Babesia vogeli* has been detected in dogs from northern Australia [17, 18] and New South Wales [18], and is transmitted by *R. sanguineus* [19, 20]. *Babesia gibsoni* has been detected in dogs from south-eastern Australia [21]. Evidence in Japan suggests that *H. longicornis*, which is also distributed in Australia, is a vector of *B. gibsoni* [22], and there is also one report of direct transmission of the piroplasm between dogs in Australia [23].

Although cytauxzoonosis is a major TBD of cats in the United States [24, 25], neither cytauxzoonosis nor any other TBD of cats are known to occur in Australia. Equine piroplasmosis was first diagnosed in Australia in 1976 [26]. The disease was later confirmed to be caused by the protozoan pathogen *Babesia equi* [27], which has since been redescribed as *Theileria equi* [28]. The presence of *T. equi* in horses in Australia was considered to have occurred due to the importation of infected horses during the twentieth Century [29], however, the disease remained localised and Australia is now free of equine piroplasmosis [29, 30].

Tick infestations can cause other health problems in companion animals. Tick paralysis manifests as ascending paralysis and local neurological deficits [31–33]. The Australian tick species known to frequently cause tick paralysis in eastern and south-eastern Australia are *I. cornuatus* and *I. holocyclus* [34]. Tick paralysis caused by *Ixodes hirsti* has also been reported in cats [35]. Additionally, heavy or repeated infestations of ticks can cause anaemia in the host animal, which is associated with blood loss during tick feeding [36]. Immunosuppression [37], secondary infections at the bite site [38], and localised dermatitis [39] can also result from tick infestations.

The present study aimed to determine the tick species that are associated with dogs, cats and horses in Australia, and is part of broader research investigating tick-borne pathogens.

## Methods

### Sample collection

Individual ticks (*n* = 4765) were collected during 2012–2015 from a total of 837 companion animal hosts (*n* = 4191 from 643 dogs; *n* = 345 from 42 horses; *n* = 229 from 152 cats) from New South Wales (NSW), the Northern Territory (NT), Queensland (QLD), South Australia (SA), Tasmania (TAS), Victoria (VIC), and Western Australia (WA). Ticks were removed from animals by staff at veterinary clinics, and by various persons throughout Australia in response to a nationwide advertising campaign. The ticks were preserved in 70 % ethanol and were sent to Murdoch University for analysis. For each submission received, the source, approximate geographic location of collection site, host, and date of collection was recorded.

### Ethics statement

The Murdoch University Animal Ethics Committee sanctioned the opportunistic removal of ticks from animal hosts. The use of questionnaires was approved by the Murdoch University Human Research Ethics Committee (Permit No. 2011/005).

### Tick identification

Individual ticks were examined with an Olympus SZ61 stereomicroscope (Olympus, Center Valley, PA, USA) with a Schott KL 1500 LED light source (Schott AG, Mainz, Germany). Photographs were taken with an Olympus SC30 digital camera and analysis getIT software (Olympus, Center Valley, PA, USA). The instar, sex, and species were morphologically identified [14, 40] and the data were recorded with Microsoft® Excel® for Mac 2011, version 14.5.2.

### Sample mapping

The sample collection locations were geo-referenced using the open source software QGIS, version 2.10.1 [41] with the latest Australian coordinate system: Geocentric Datum of Australia 1994 (GDA94) [42]. Layers were styled with a categorised renderer, with layer symbology classified according to tick species, and a point displacement renderer was used to visualise overlapping points around a centre symbol on rendering circles [43].

### Questionnaire design

A questionnaire was designed in conjunction with Bayer Australia Ltd to obtain information about the age, sex, weight, habitat, use of tick control products and clinical signs of tick paralysis [44] of dogs, cats and horses that were presented to veterinary clinics (Additional file 1). The companion animal owners completed the questionnaires while at the veterinary clinic. A total of 433 questionnaires from 30 veterinary clinics were collected by Bayer Australia Ltd area managers, and sent to Murdoch University.

### Statistical analysis

The database of tick identification results, sample information, and questionnaire data was generated and summarised with Microsoft® Excel® for Mac 2011, version 14.5.2, and IBM® SPSS® Statistics 2013 software, version 22 (Armonk, NY, USA). During the analysis, dogs were considered small if their weight was ≤ 10 kg, medium if 11–19 kg, and large if ≥ 20 kg, and the scale of tick paralysis was recoded into a binary variable (present or absent).

## Results

### Morphological identification of Ixodidae

Overall, 220 larvae, 805 nymphs, 1404 males, and 2336 females were identified from 837 companion animals in 7/8 Australian states and territories. The number and location (state) of ixodids that were identified on dogs, cats, and horses are presented in Tables 2, 3 and 4. Photographs of a single female for each species identified, except for *Bothriocroton* sp., are displayed in Additional file 2.

**Table 2:** Tick species, location and number of instars collected from dogs

		Number of instars					
Species	State	Larvae	Nymphs	Males	Females	Instar total	Number of hosts
*Amblyomma triguttatum triguttatum*	WA	0	5	0	5	10	8
*Bothriocroton* sp.	TAS	0	1	0	0	1	1
	VIC	11	0	1	1	13	5
*Haemaphysalis bancrofti*	NSW	0	2	0	2	4	3
	QLD	0	0	0	1	1	1
*Haemaphysalis longicornis*	NSW	0	127	0	61	188	35
	QLD	2	23	0	0	25	4
*Ixodes cornuatus*	TAS	0	7	0	6	13	11
	VIC	0	2	0	0	2	2
*Ixodes holocyclus*	NSW	0	39	78	373	490	193
	QLD	0	47	4	226	277	205
	TAS	0	0	0	2	2	2
	WA	0	0	0	1	1	1
*Ixodes myrmecobii*	WA	0	0	0	4	4	4
*Ixodes tasmani*	NSW	0	0	0	1	1	1
	QLD	0	0	0	4	4	2
	TAS	16	4	6	57	83	49
	VIC	0	0	0	2	2	2
*Rhipicephalus australis*	QLD	0	1	0	0	1	1
*Rhipicephalus sanguineus*	NSW	0	0	0	6	6	3
	NT	31	240	927	858	2056	76
	QLD	0	12	22	32	66	15
	SA	5	55	231	119	410	15
	WA	132	168	115	116	531	46
Total		197	733	1384	1877	4191	685^a^

^a^The total number of dogs sampled in this study was *n* = 643. The total number of host records presented in Table 2 (*n* = 685) is inflated due repeated measures that occurred in cases where more than one tick species was identified on a host

**Table 3:** Tick species, location and number of instars collected from cats

		Number of instars					
Species	State	Larvae	Nymphs	Males	Females	Instar total	Number of hosts
*Haemaphysalis bancrofti*	NSW	0	0	0	1	1	1
*Ixodes cornuatus*	TAS	0	0	0	1	1	1
*Ixodes hirsti*	TAS	0	0	0	1	1	1
*Ixodes holocyclus*	NSW	0	22	9	88	119	77
	QLD	0	2	0	64	66	59
*Ixodes myrmecobii*	WA	0	0	0	1	1	1
*Ixodes tasmani*	TAS	23	8	0	7	38	11
	VIC	0	1	0	0	1	1
*Rhipicephalus sanguineus*	NSW	0	0	0	1	1	1
Total		23	33	9	164	229	153^a^

^a^The total number of cats sampled in this study was *n* = 152. The total number of host records presented in Table 3 (*n* = 153) is inflated due repeated measures that occurred in cases where more than one tick species was identified on a host

**Table 4:** Tick species, location and number of instars collected from horses

		Number of instars					
Species	State	Larvae	Nymphs	Males	Females	Instar total	Number of hosts
*Amblyomma triguttatum triguttatum*	NSW	0	0	0	1	1	1
	QLD	0	0	0	4	4	1
	WA	0	1	0	9	10	8
*Haemaphysalis bancrofti*	NSW	0	1	2	10	13	3
	QLD	0	2	1	6	9	5
*Haemaphysalis longicornis*	NSW	0	27	0	176	203	14
	QLD	0	2	0	2	4	2
*Ixodes holocyclus*	NSW	0	5	2	33	40	11
	QLD	0	0	6	51	57	10
*Ixodes tasmani*	QLD	0	0	0	1	1	1
*Rhipicephalus australis*	QLD	0	1	0	2	3	1
Total		0	39	11	295	345	57^a^

^a^ The total number of horses sampled in this study was *n* = 42. The total number of host records presented in Table 4 (*n* = 57) is inflated due repeated measures that occurred in cases where more than one tick species was identified on a host

### Host records

One novel host record was obtained for *I. myrmecobii*; one female *I. myrmecobii* was collected from *Felis catus* (domestic cat) in the town of Esperance, WA (Additional file 3). All other host records of dogs, cats, and horses for the various tick species identified were consistent with previous host records (Table 1).

### Ixodidae collection locations

**Fig. 1:**
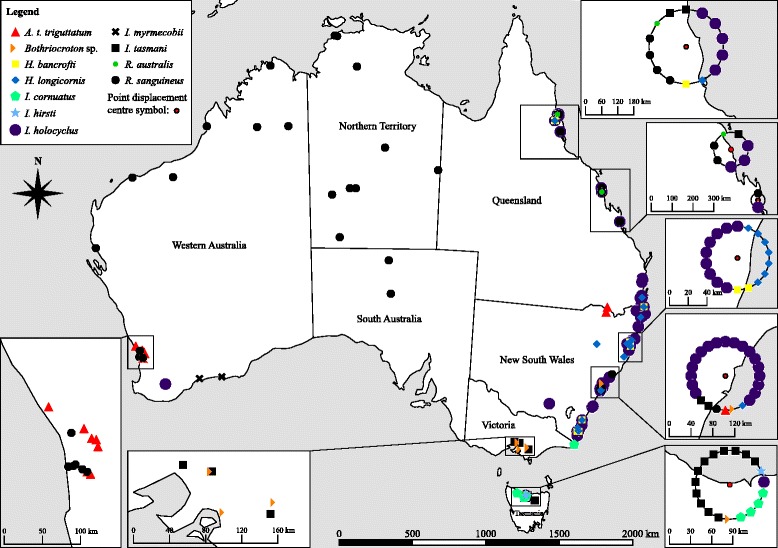
Collection locations of ticks removed from dogs, cats, and horses in Australia. Each point represents a unique collection location for the corresponding tick species. Overlapping points were displaced with a point displacement renderer around a centre symbol (denoted in legend); point displacement distance was defined by number of map units (kilometres)

The collection locations for each ixodid species identified from companion animal hosts are presented in Fig. 1.

The individual geographic collection locations, including the coordinates that were geo-referenced and displayed in Fig. 1, for the instars identified on dogs, cats, and horses are listed in Additional file 3. Several collection locations occurred outside of the previously recorded distribution ranges for the following species: *H. longicornis* (one in the suburb of Sancrox, NSW) [14]; *I. holocyclus* (two in TAS, and one in WA) [40]; and *R. sanguineus* (72 in southwest WA, 17 canine hosts; 410 in SA, 15 canine hosts) [45].

### Questionnaires

**Fig. 2:**
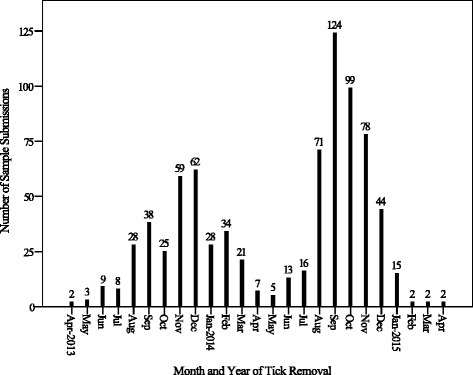
Number of sample submissions in each month from 2013–2015

The majority of samples were received in the years 2013 (28 %) and 2014 (65 %), and most were collected from companion animal hosts during the months of spring (September-November) and summer (December-February) (Fig. 2). The data gained from responses to questionnaires is summarised in Table 5.

**Table 5:** Summary of questionnaire responses

Host factors		Hosts		
		Dogs	Cats	Horses
Sex	Female	152	47	3
	Male	167	55	4
	Total	319	102	7
Age (months)	Median	60	36	127
	Interquartile range	24–108	19.5–96	90–240
	Minimum	2	1	72
	Maximum	240	244	300
	Total	311	98	7
Clinical signs of tick paralysis	Absent	65	9	5
	Present	153	77	2
	Total	218	86	7
Are tick control products used?	No	106	56	2
	Yes	168	42	4
	Total	274	98	6
Dog Size	Small	139	N/A	N/A
	Medium	53	N/A	N/A
	Large	120	N/A	N/A
	Total	312	N/A	N/A
Habitat	Indoors	114	N/A	N/A
	Outdoors	135	N/A	N/A
	Total	249	N/A	N/A
Where is dog walked?	Confined to home	29	N/A	N/A
	Urban parks	73	N/A	N/A
	Semi-urban bush	38	N/A	N/A
	Countryside/remote bush/farmland	35	N/A	N/A
	Urban parks and semi-urban bush	15	N/A	N/A
	Urban parks, semi-urban bush and countryside/remote bush/farmland	8	N/A	N/A
	Semi-urban bush and countryside/remote bush/farmland	18	N/A	N/A
	Urban parks and countryside/remote bush/farmland	1	N/A	N/A
	Total	217	N/A	N/A

In the vast majority of cases where signs of tick paralysis were reported in companion animals, *I. holocyclus* was identified from the host (97 %; 226/232) (data not shown). In one case, *R. sanguineus* was removed from a cat with tick paralysis. The remaining five cases of tick paralysis were reported in dogs that were infested with *Bothriocroton* sp. (*n* = 1), *I. tasmani* (*n* = 1), *Haemaphysalis bancrofti* (*n* = 1) and *R. sanguineus* (*n* = 2) (data not shown).

## Discussion

This report describes the first comprehensive nationwide survey of ticks associated with companion animals in Australia and the results are generally consistent with the individual geographical distributions and host records [14, 40, 46–54], with a few exceptions. Interestingly, one novel host record was obtained in this study for *I. myrmecobii* on *F. catus* in Esperance, WA. Although native Australian ticks primarily feed on native wildlife species [14], they also feed on a variety of introduced mammals and birds [46–58]. The primary hosts of the introduced species *H. longicornis* and *R. australis* are cattle, but these ticks have been recorded on other livestock, introduced and native wildlife, and companion animals [14, 54, 58].

The collection locations obtained for the vast majority of ticks in this study adhered to previously described Australian distribution ranges, or to previous collection locations [14, 40, 45, 47, 52, 54, 59]. The records of two *I. holocyclus* in TAS, and one *I. holocyclus* in the city of Wagga Wagga, NSW, most likely occurred due to travel to *I. holocyclus* endemic areas [40] prior to tick removal, which was documented by the companion animal owners. Given that the distribution of ticks is affected by climate, vegetation, and the presence of the primary host species [60], it is also likely that the single *I. holocyclus* recorded from a dog in southwest WA is a result of interstate travel from *I. holocyclus* endemic areas. The collection locations that occurred outside of the previously recorded distribution ranges for *H. longicornis* and *R. sanguineus* [14, 45] may also be attributable to travel, since people and their companion animals can readily travel with, and potentially disperse, ticks outside of their endemic range.

It is probable that the distribution of *R. sanguineus* has extended further south of the NT border into northern and central SA, which is comprised of the same terrestrial ecoregion as southern and central NT (deserts and xeric shrub lands) [61]. Investigations of *R. sanguineus* group ticks overseas have found two paraphyletic lineages of *R. sanguineus*: the tropical (northern) lineage [*R. sanguineus* (*sensu lato*)]; and the temperate (southern) lineage [possibly *R. sanguineus* (*sensu stricto*)] [62–66], and these lineages may represent two different species [66]. These paraphyletic groupings remain to be investigated across different climatic regions of Australia.

The collection localities of *I. myrmecobii* along the southern coastline of WA obtained in this study are novel. The information pertaining to the distribution range of this enigmatic tick species is limited, with very few studies of *I. myrmecobii* conducted [14, 67, 68]. Formal geographical distribution data for many of the Australian tick species we report in this study is either non-existent, or requires a systematic study.

*Bothriocroton* ticks collected from dogs in TAS and VIC (*n* = 10) could not be identified to the species level as the morphological features were too damaged in the male and female specimens, and there is currently no key for the identification of *Bothriocroton* nymphal and larval species. These specimens are likely *Bothriocroton auruginans*, which is distributed in TAS and VIC [14, 40], and is the only species of *Bothriocroton* that parasitises dogs in Australia. The current Australian tick morphology keys [14, 40] also lack a complete description of *I. cornuatus* instars; therefore, the *I. cornuatus* nymphs examined in this study have been only tentatively identified, pending further species confirmation by molecular techniques.

There were no male *H. longicornis* ticks identified in this study, which was expected, as the populations of *H. longicornis* in Australia (as well as in north-eastern Russia, northern Japan, New Zealand, New Caledonia, and Fiji) are parthenogenetic [69], and represent the only known example of triploidy in ticks [70]. In Australia, very few males have ever been reported [50, 71].

The use of standard Australian tick morphology keys to identify ticks collected in Australia seems appropriate given the context of the study, however, there are species found elsewhere with similar morphology to those that are present in Australia. It is possible that other tick species could be inadvertently introduced into this country as a result of international movements of animals and humans, thus future studies could include molecular phylogenetic analyses of genetic markers (e.g. mitochondrial cytochrome *c* oxidase subunit 1 (*cox*1), 12S ribosomal RNA (rRNA), and 16S rRNA genes) to increase the confidence and accuracy of tick identification.

As expected, the majority of the ticks examined in this study were collected during the warmer months of spring and summer, when ixodids are generally more abundant [72–76] (Fig. 2). There is limited data pertaining to ownership of companion animals in Australia. A 2013 survey of 1089 pet owners reported that 76 % of dogs are kept exclusively or partly indoors [10]. Conversely in this study, 54 % of dogs usually lived outdoors (i.e. in a kennel), and 13 % were confined to the home. Increased exposure to tick habitats likely increases the chance of tick attachment, which could explain our observations, as only dogs with ticks were sampled in this study. Overseas studies have reported that factors such as host species, breed, and habitat significantly affect the likelihood of tick species attachment [75, 77]. Explanatory variables for tick species attachment in this study could not be fairly assessed, as questionnaire data was skewed towards companion animals that were infested with *I. holocyclus* on the eastern coast of Australia.

Several tick species identified in this study are of potential concern to the health of companion animals according to the current literature. Importantly, *R. sanguineus* is a well-known vector of *B. vogeli* [78], the cause of canine babesiosis. Most of the animals that were infested with *I. holocyclus* had clinical signs of tick paralysis (77 %; 226/293) (data not shown), and this condition can be fatal [79, 80]. The reports of tick paralysis in one cat infested with *R. sanguineus*, and in five dogs infested with *Bothriocroton* sp., *I. tasmani*, *H. bancrofti* and *R. sanguineus* are unusual. These may have been reported erroneously on the questionnaire, or *Ixodes* spp. known to cause tick paralysis might have attached to the animal, but were not collected.

There are few reports of TBD associated with *I. cornuatus* [81] and virtually nothing is known about pathogens transmitted by *I. myrmecobii* and *I. hirsti*. Although *Amblyomma triguttatum triguttatum*, *H. bancrofti*, *H. longicornis*, *I. tasmani* and *R. australis* have been associated with TBD in other host species [40, 82–87], it remains to be investigated whether these species carry pathogens that could impact the health of companion animals.

## Conclusions

This first nationwide study of ticks on companion animals in Australia has provided a comprehensive snapshot of the current tick-host associations in dogs, cats, and horses that should be of interest to pet owners and carers, veterinarians, and manufacturers of ectoparasiticides. The species that were most commonly found on these animals are well-known vectors of pathogens, or cause neurological disease. However, the vector competency of several species identified has not been widely investigated. Such knowledge is required to better understand the risks of TBD transmission to pets and potentially, to their owners. Further investigations are required to establish the environmental and host factors that influence tick species infestations on companion animals, which may help to develop prevention strategies against tick infestations.

## Additional files

Additional file 1: Questions answered by companion animal owners at veterinary clinics. (DOCX 55 kb) Additional file 2: Dorsal and ventral photographs of female ixodids. A) Dorsal view of *Amblyomma triguttatum triguttatum*. B) Ventral view of *A. t. triguttatum*. C) Dorsal view of *Haemaphysalis bancrofti*. D) Ventral view of *H. bancrofti*. E) Dorsal view of *Haemaphysalis longicornis*. F) Ventral view of *H. longicornis*. G) Dorsal view of *Ixodes cornuatus*. H) Ventral view of *I. cornuatus*. I) Dorsal view of *Ixodes hirsti*. J) Ventral view of *I. hirsti*. K) Dorsal view of *Ixodes myrmecobii*. L) Ventral view of *I. myrmecobii*. M) Dorsal view of *Ixodes holocyclus*. N) Ventral view of *I. holocyclus*. O) Dorsal view of *Ixodes tasmani*. P) Ventral view of *I. tasmani*. Q) Dorsal view of *Rhipicephalus australis*. R) Ventral view of *R. australis*. S) Dorsal view of *Rhipicephalus sanguineus*. T) Ventral view of *R. sanguineus*. Individual tick specimens were collected from the following localities: the township of Gidgegannup, WA (A-B); the town of Missabotti, NSW (C-D); the town of Bellingen, NSW (E-F); the city of Devonport, TAS (G-J, O-P); the town of Esperance, WA (K-L); the town of Byangum, NSW (M-N); the town of Sarina, QLD (Q-R); the Indigenous Australian community of Mutitjulu, NT (S-T). (PDF 1501 kb) Additional file 3: Collection localities and number of ticks (Acari: Ixodidae) recorded on dogs (*Canis lupus familiaris*), cats (*Felis catus*), and horses (*Equus ferus caballus*). (PDF 241 kb)
