# Supplementary material for: A survey of ticks (Acari: Ixodidae) of companion animals in Australia
Source: Parasit Vectors. 2016 May 10;9:207. doi: 10.1186/s13071-016-1480-y (PMC4862205; doi:10.1186/s13071-016-1480-y)
Supplement: Additional file 1: — Questions answered by companion animal owners at veterinary clinics. (DOCX 55 kb) [file 13071_2016_1480_MOESM1_ESM.docx]

**Additional file 1**

Questions answered by companion animal owners at veterinary clinics

| **Specific questions** | **Response options** |
| --- | --- |
| Does your dog usually live outdoors (kennels)? | No; yes |
| Where do you normally walk your dog?^a^ | Only at home (confined at home); urban parks, ovals, dog exercise areas (i.e. areas maintained (e.g. mowing) by local council); semi-urban bush/scrub (i.e. local areas that are not maintained by local council); countryside/remote bush/farmland |
| Is your dog/cat/horse showing signs of tick paralysis? | No; mild; moderate; severe |
| Do you use tick control products on your dog/cat/horse? | No; yes |

^a^ One or more options could be selected.
