# Supplementary figures and images for: A survey of ticks (Acari: Ixodidae) of companion animals in Australia
Source: Parasit Vectors. 2016 May 10;9:207. doi: 10.1186/s13071-016-1480-y (PMC4862205; doi:10.1186/s13071-016-1480-y)

A

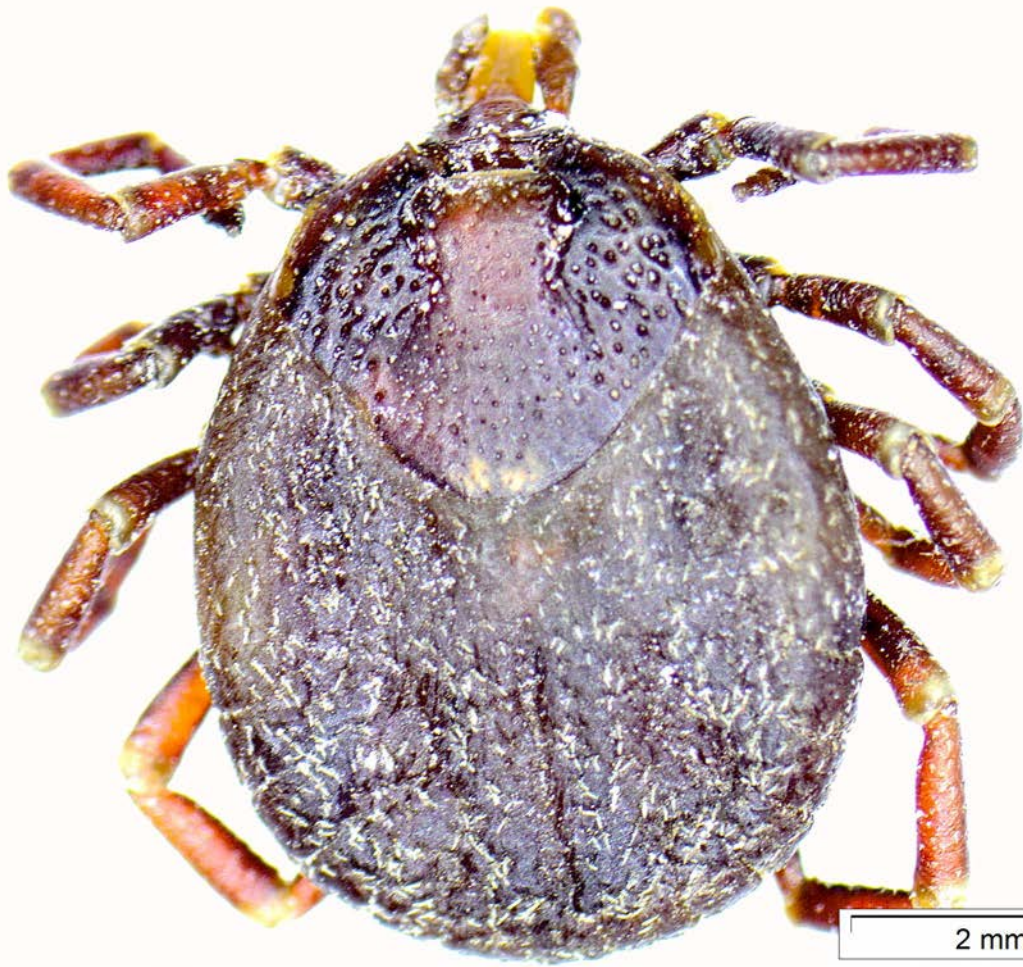

**B**

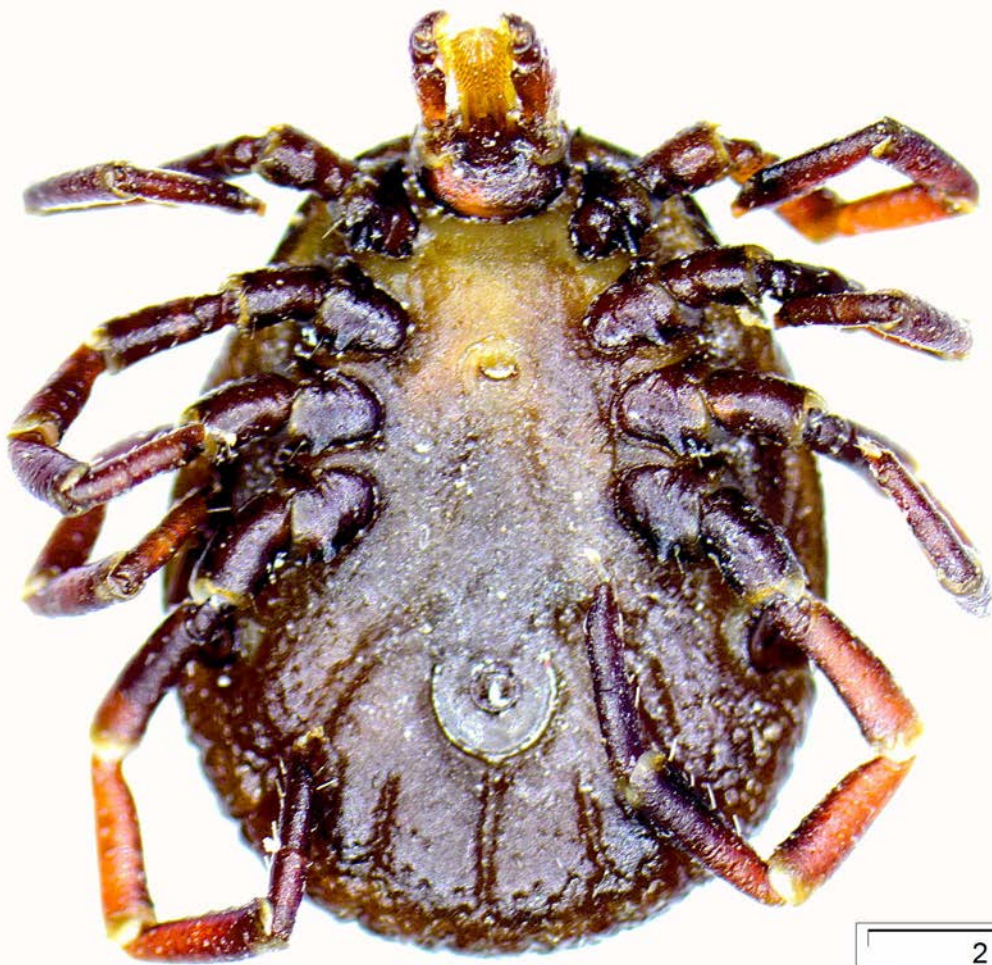

2 mm

C

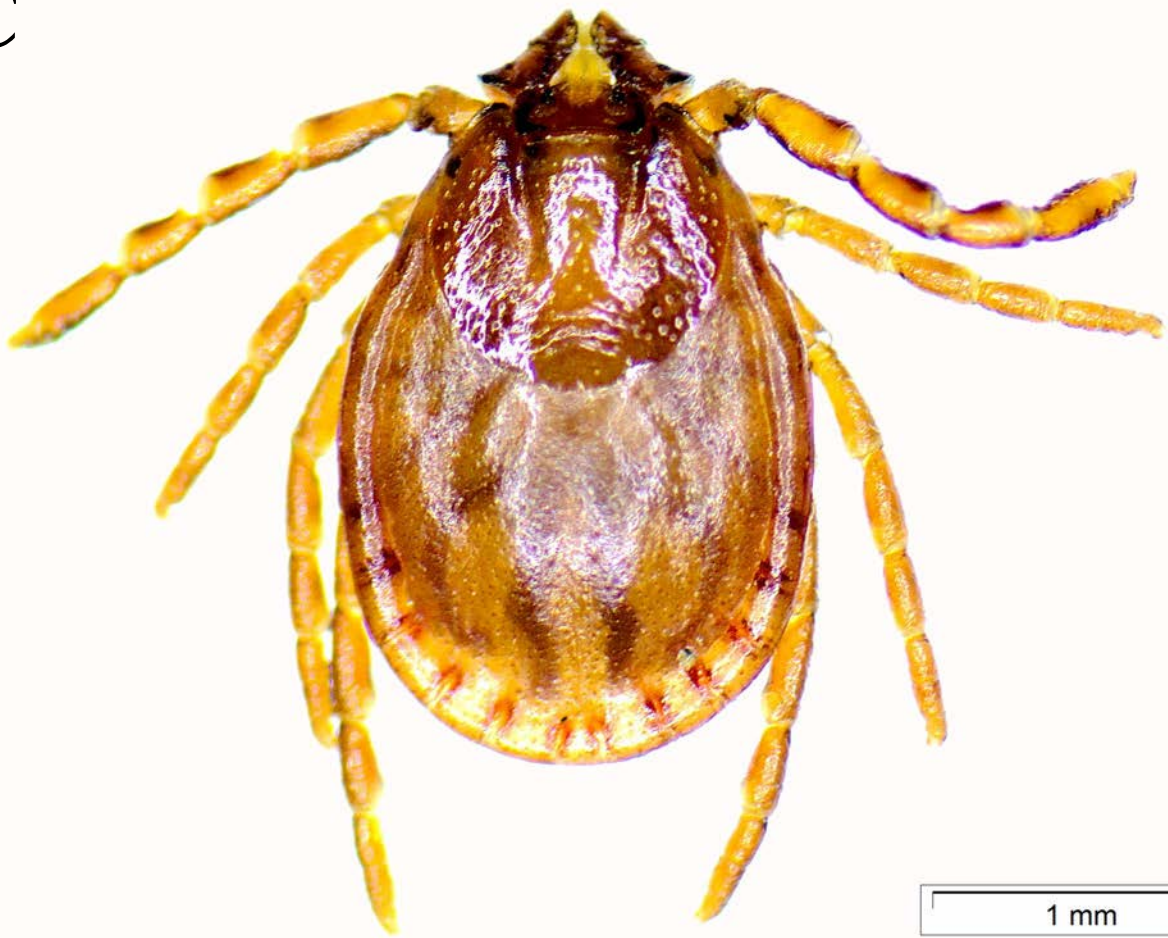

**D**

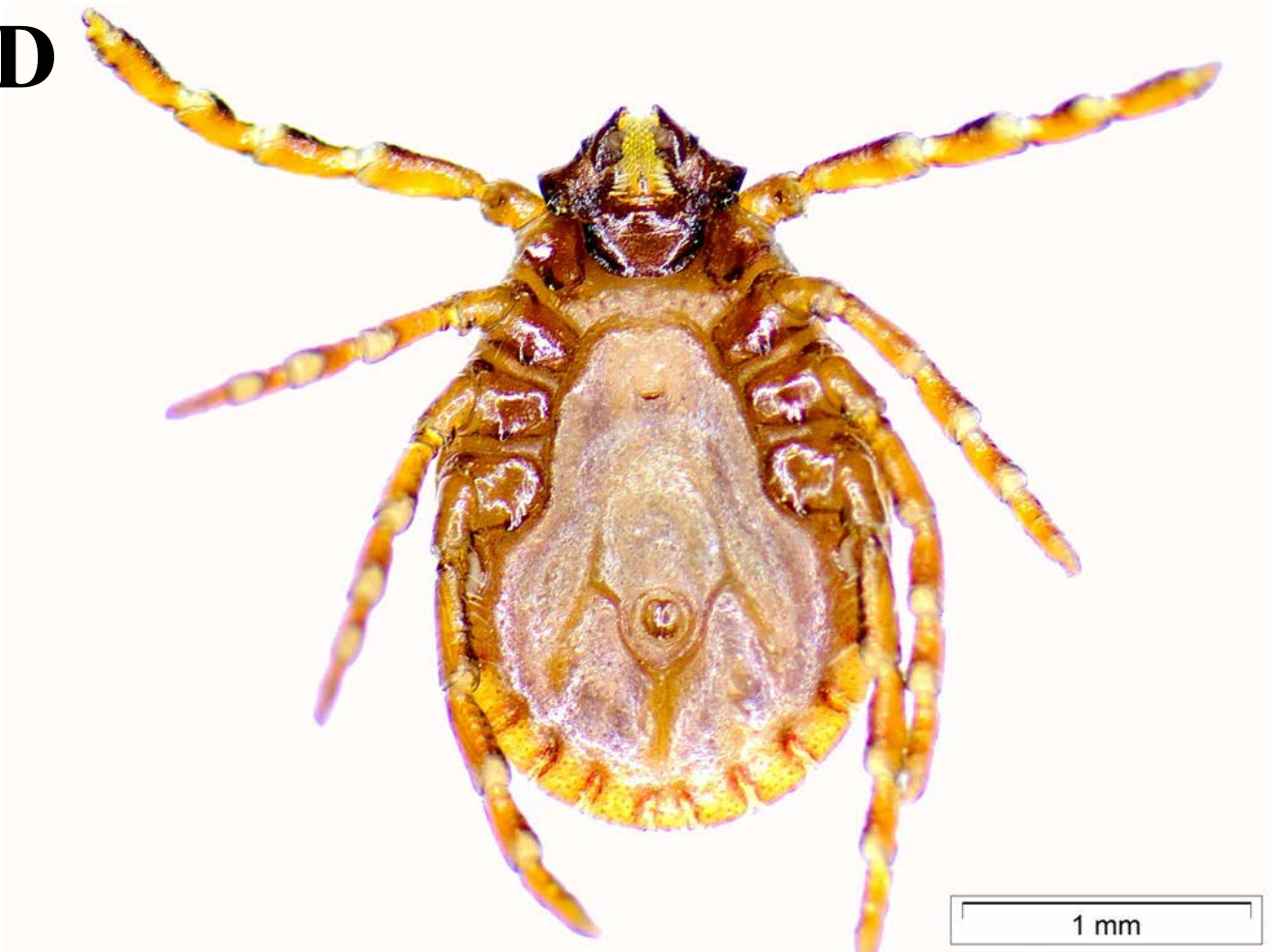

**E**

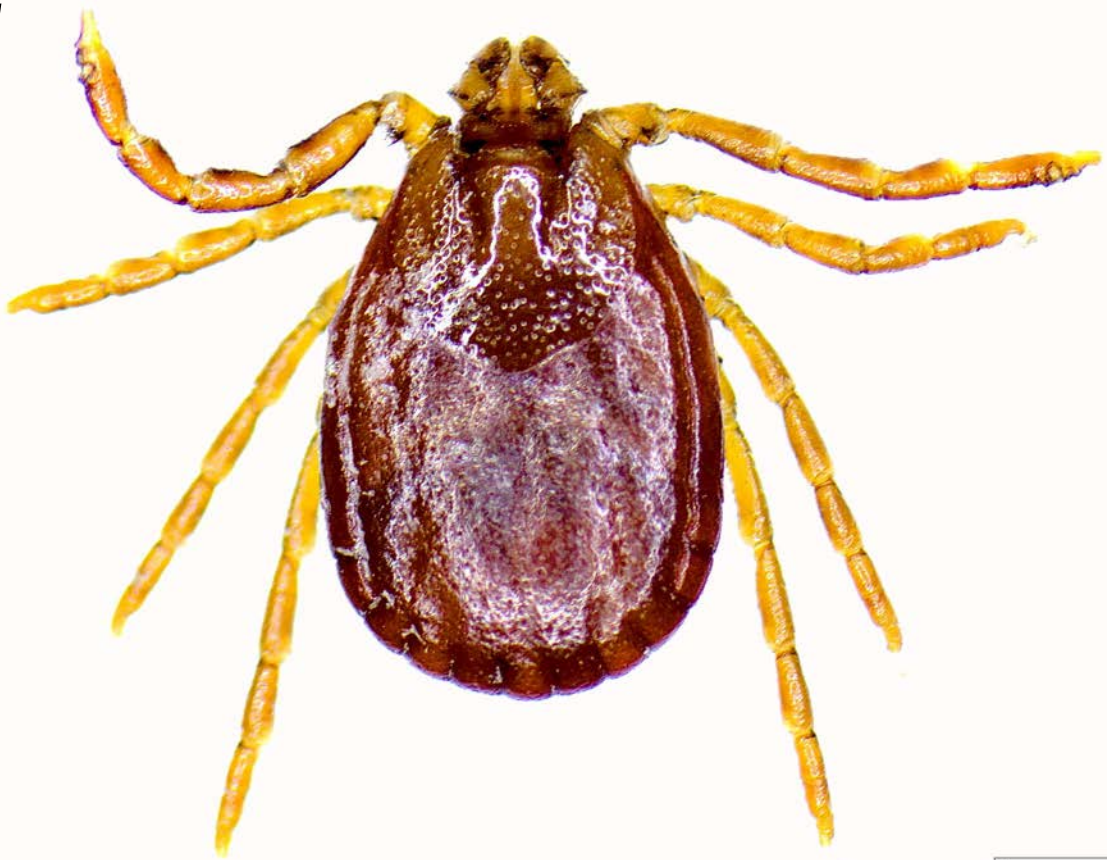

1 mm

**F**

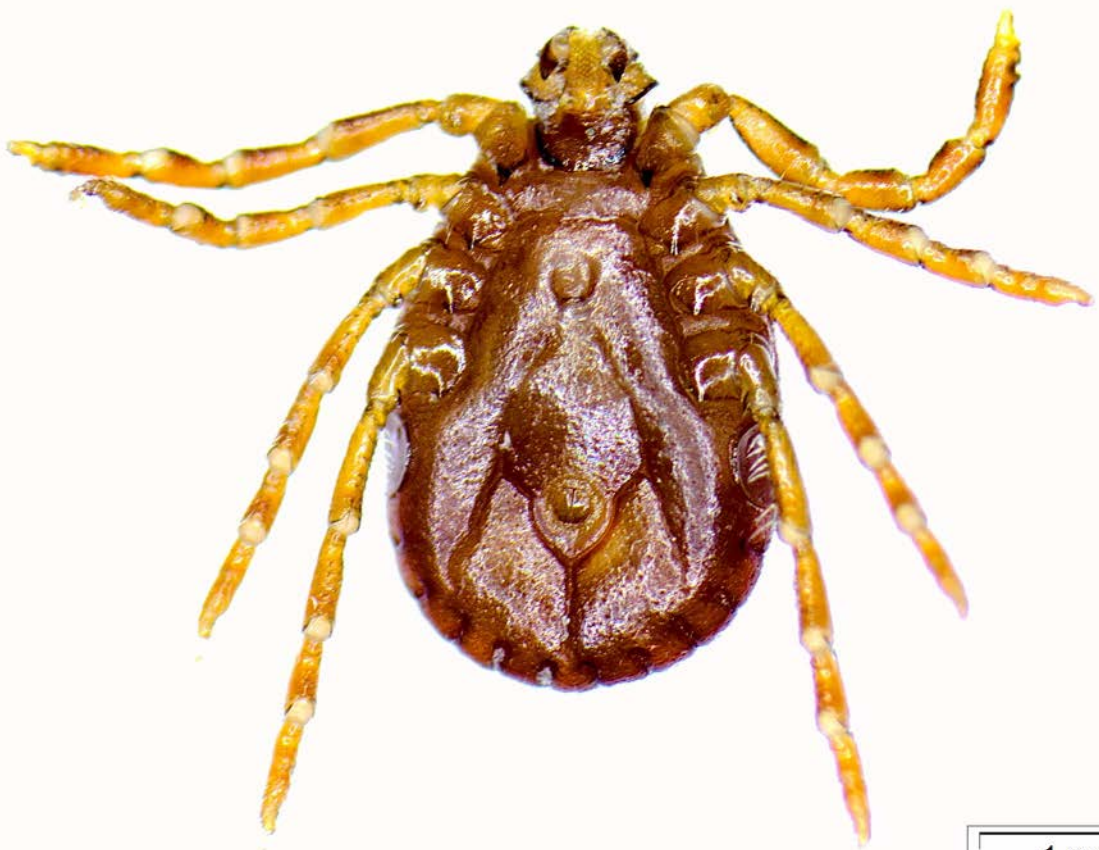

G

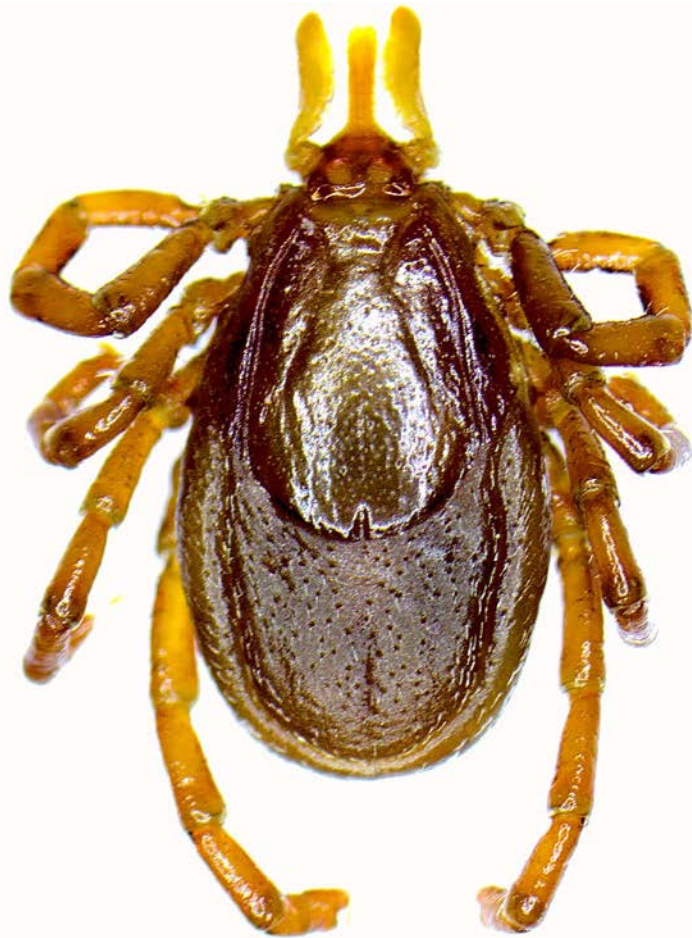

2 mm

H

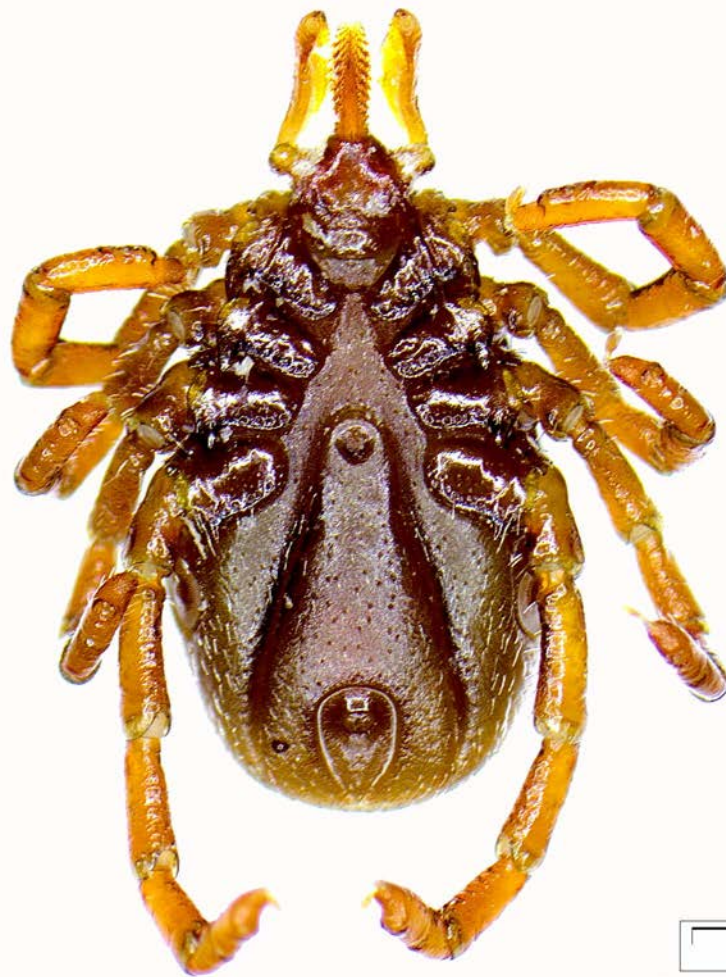

I

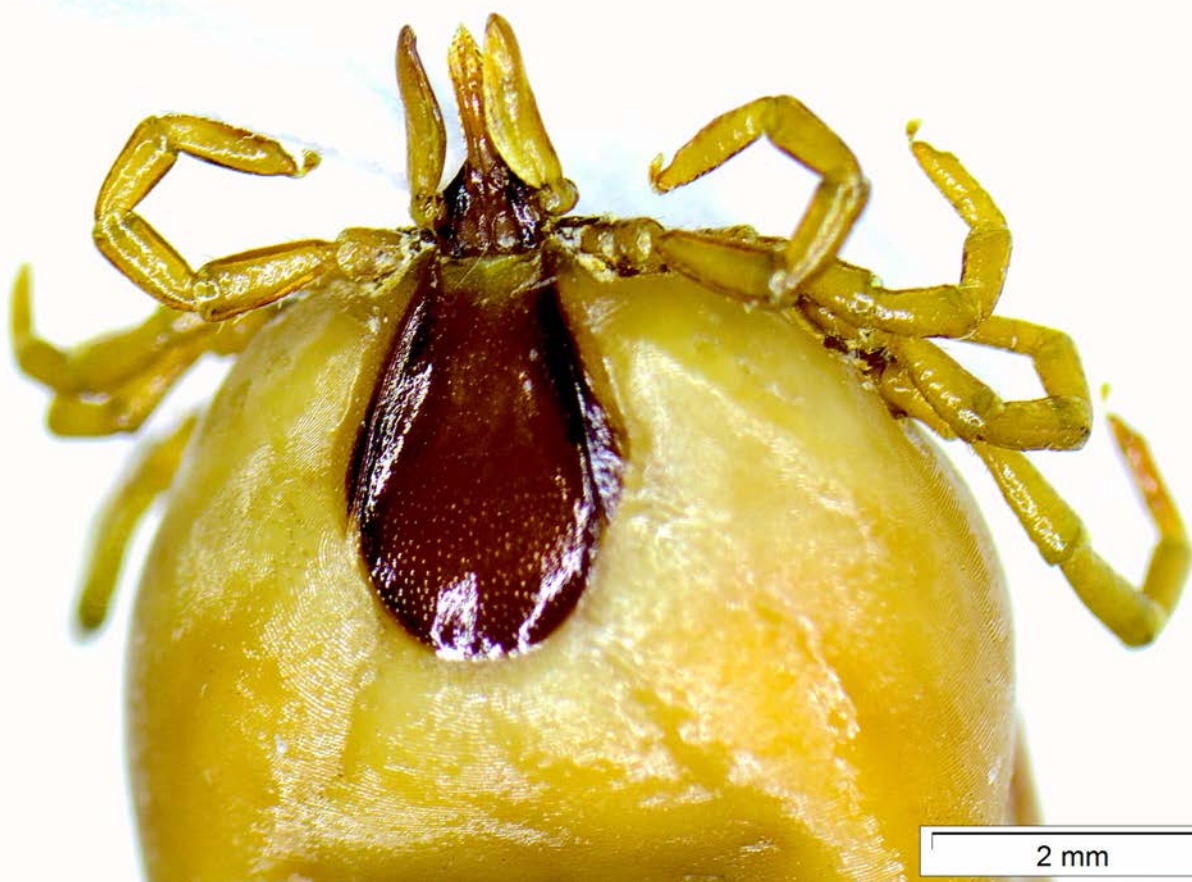

**J**

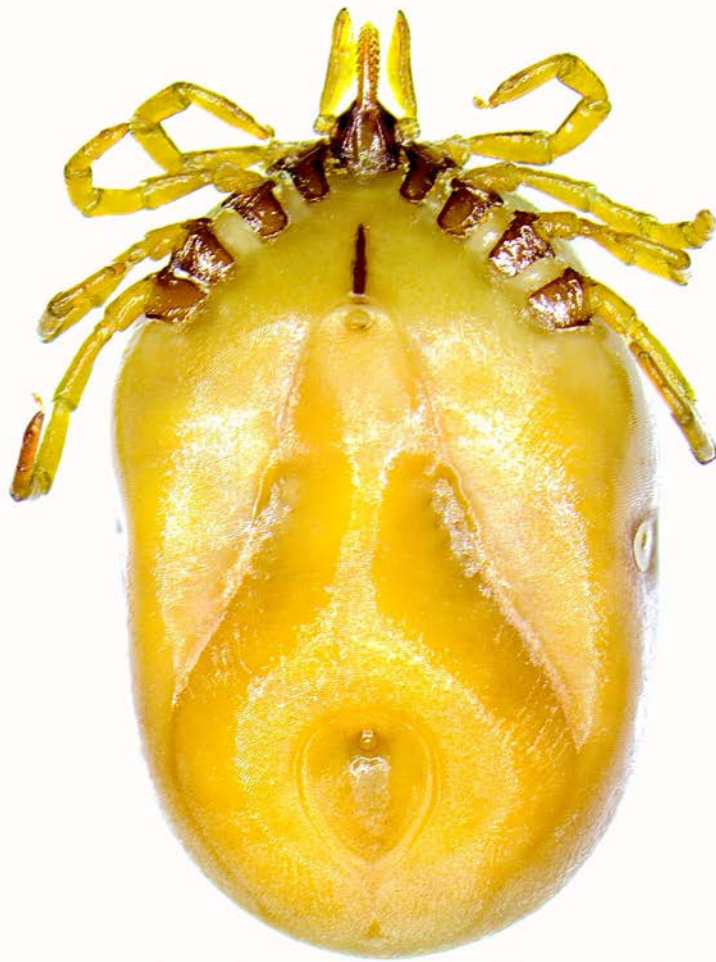

2 mm

**K**

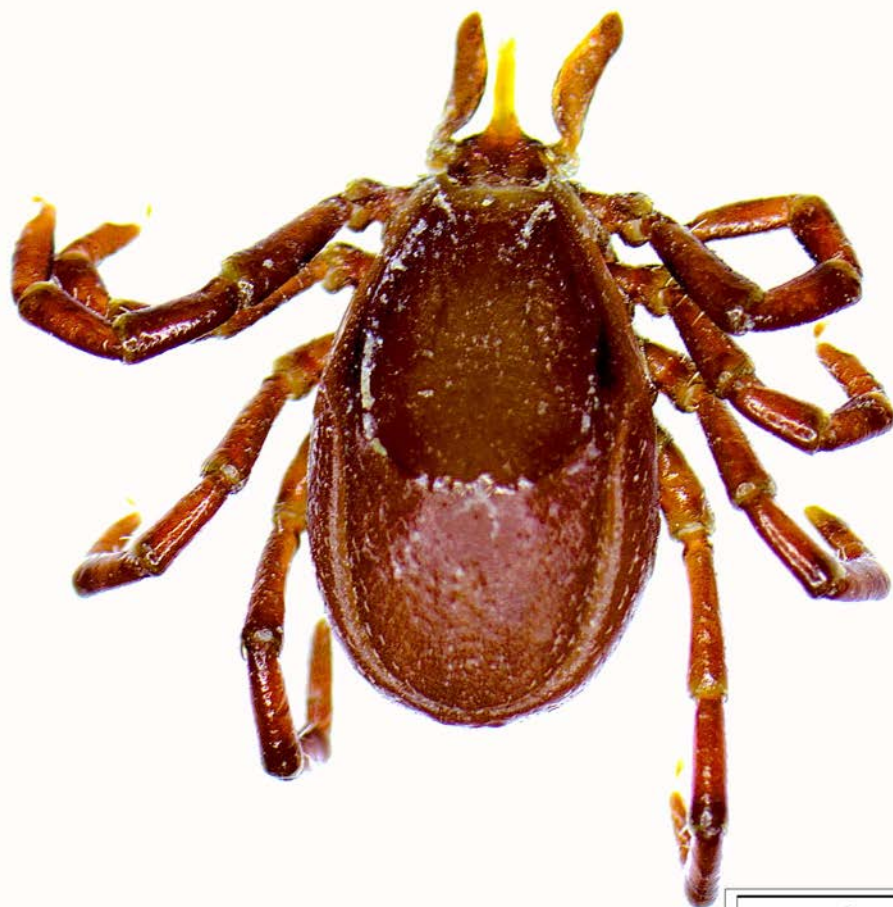

2 mm

L

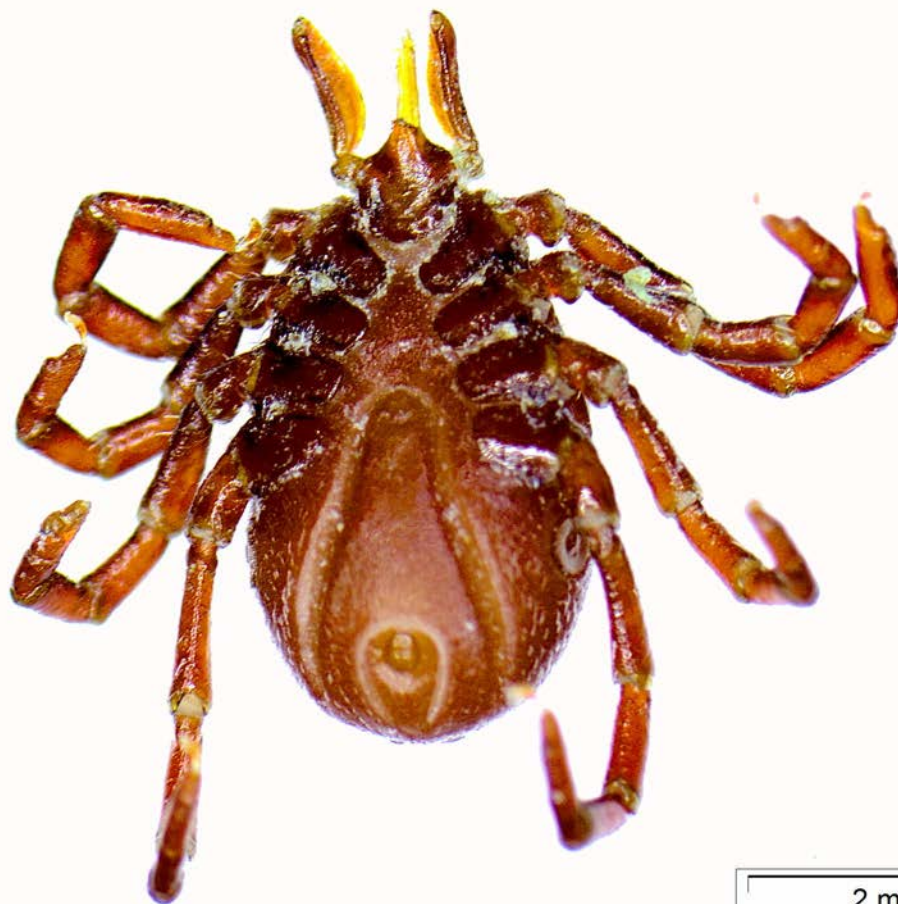

2 mm

M

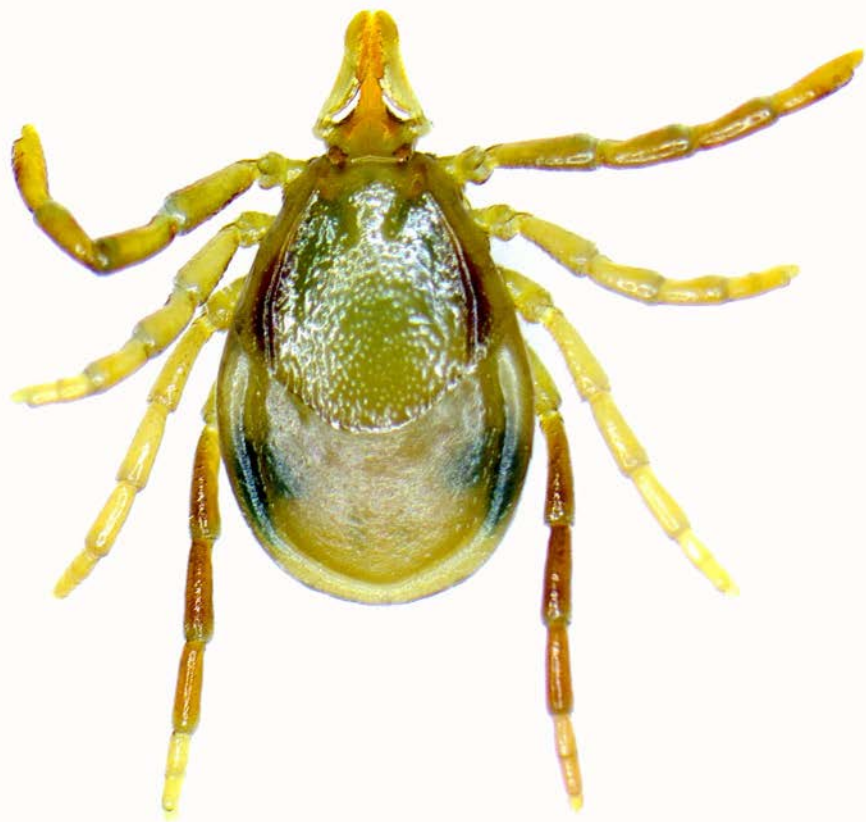

500 mm

N

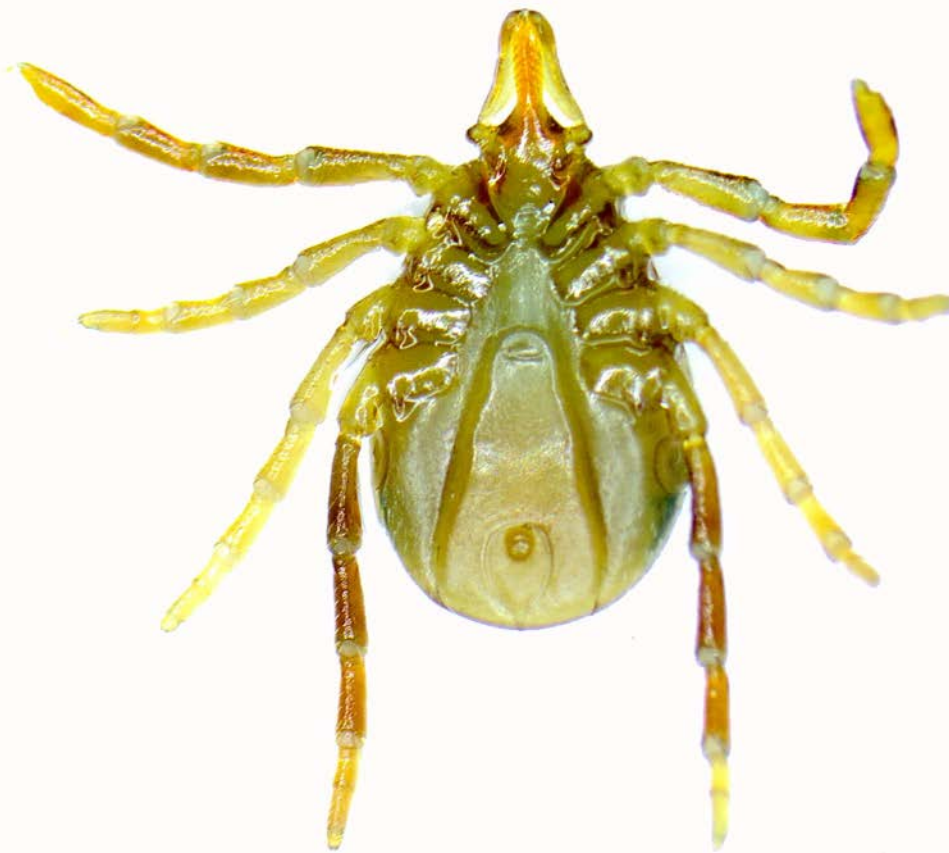

2 mm

O

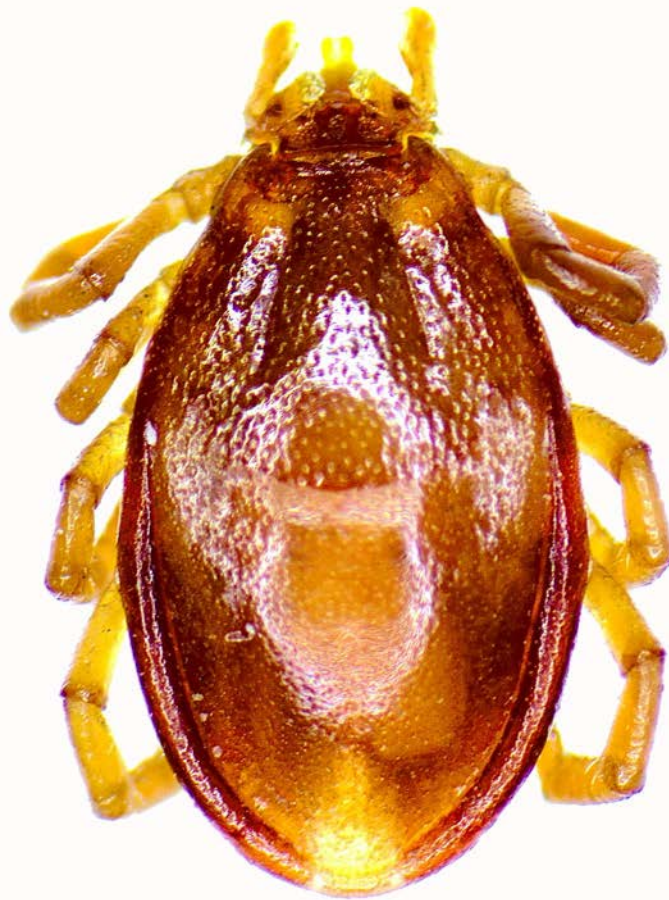

1 mm

P

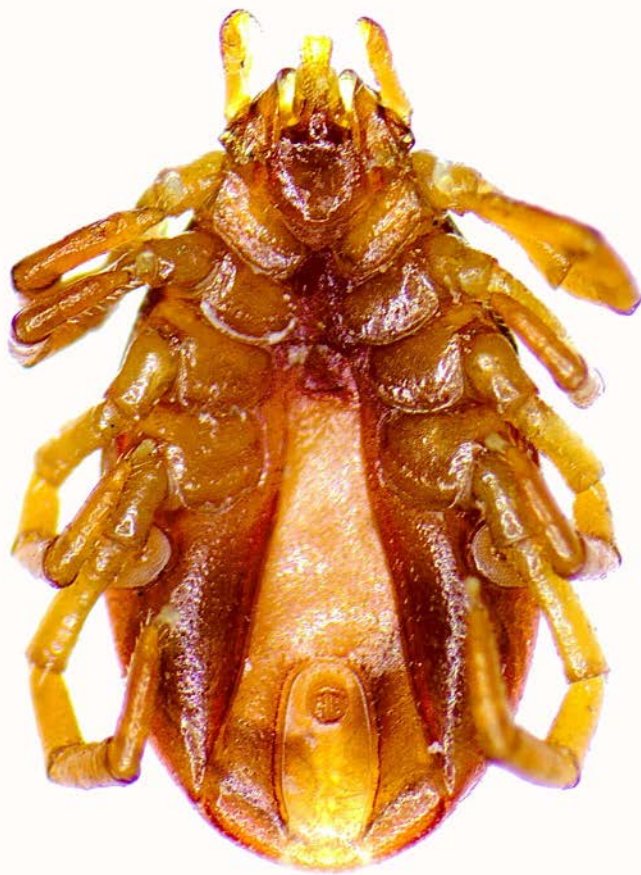

1 mm

Q

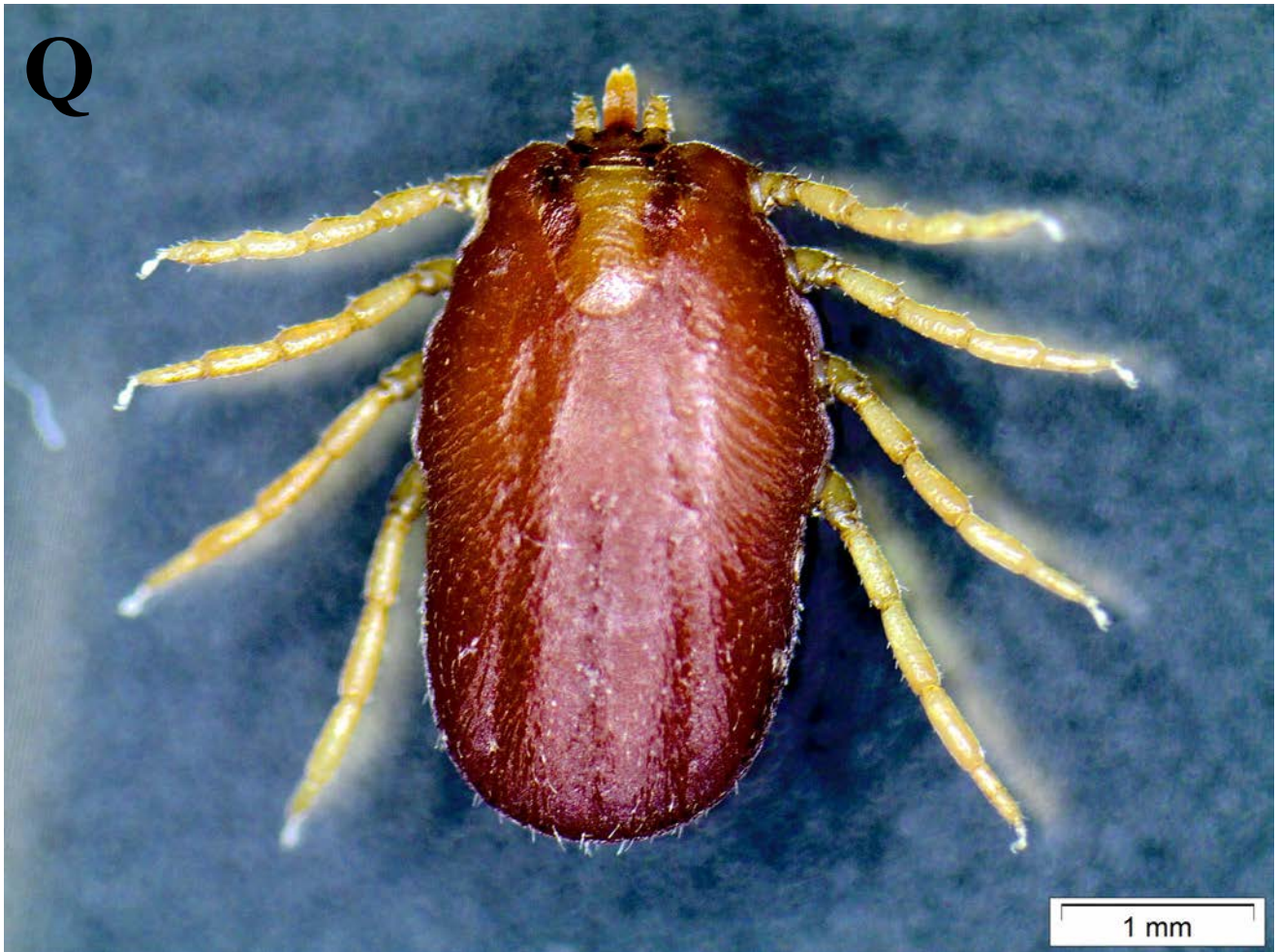

R

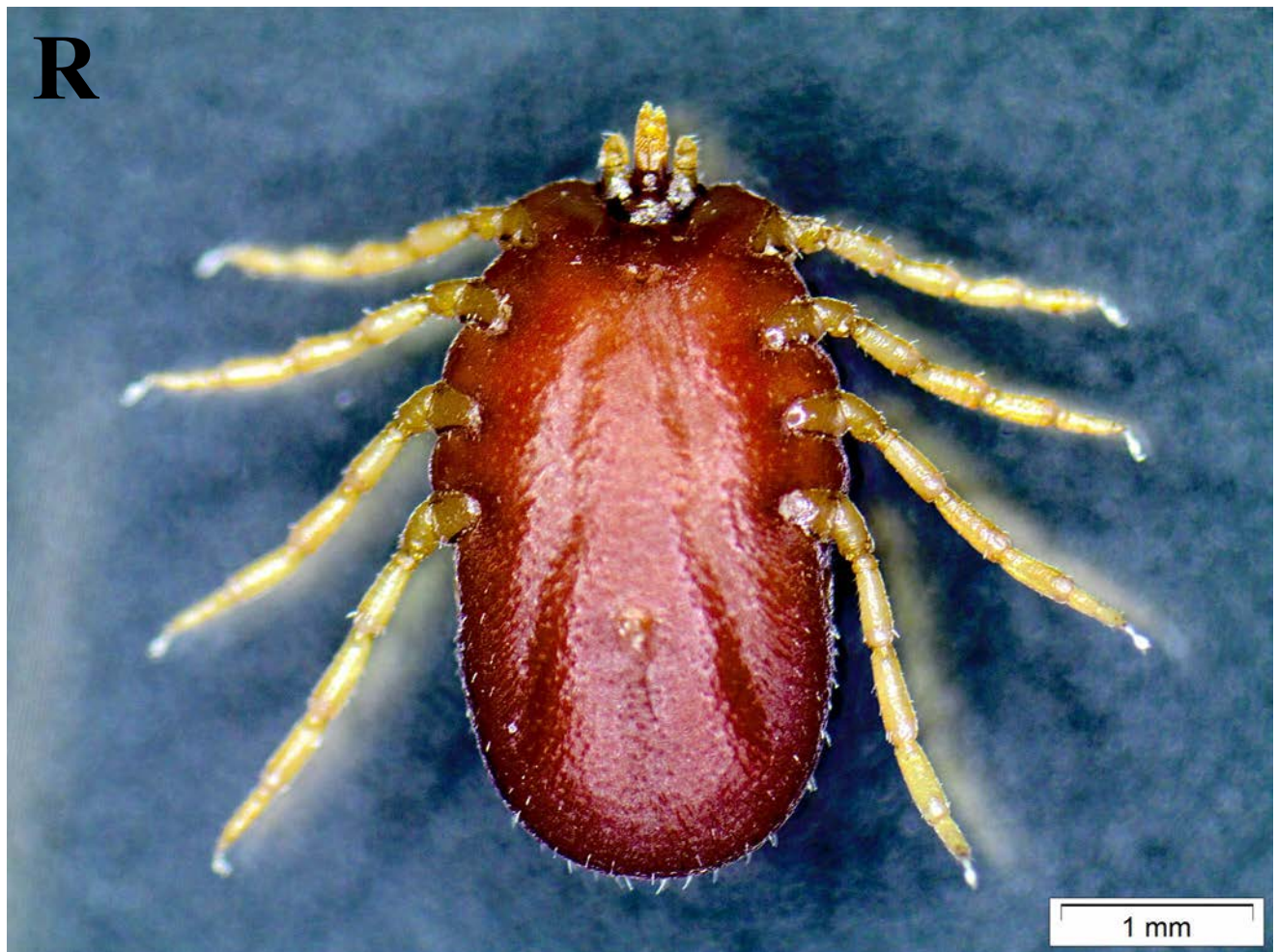

S

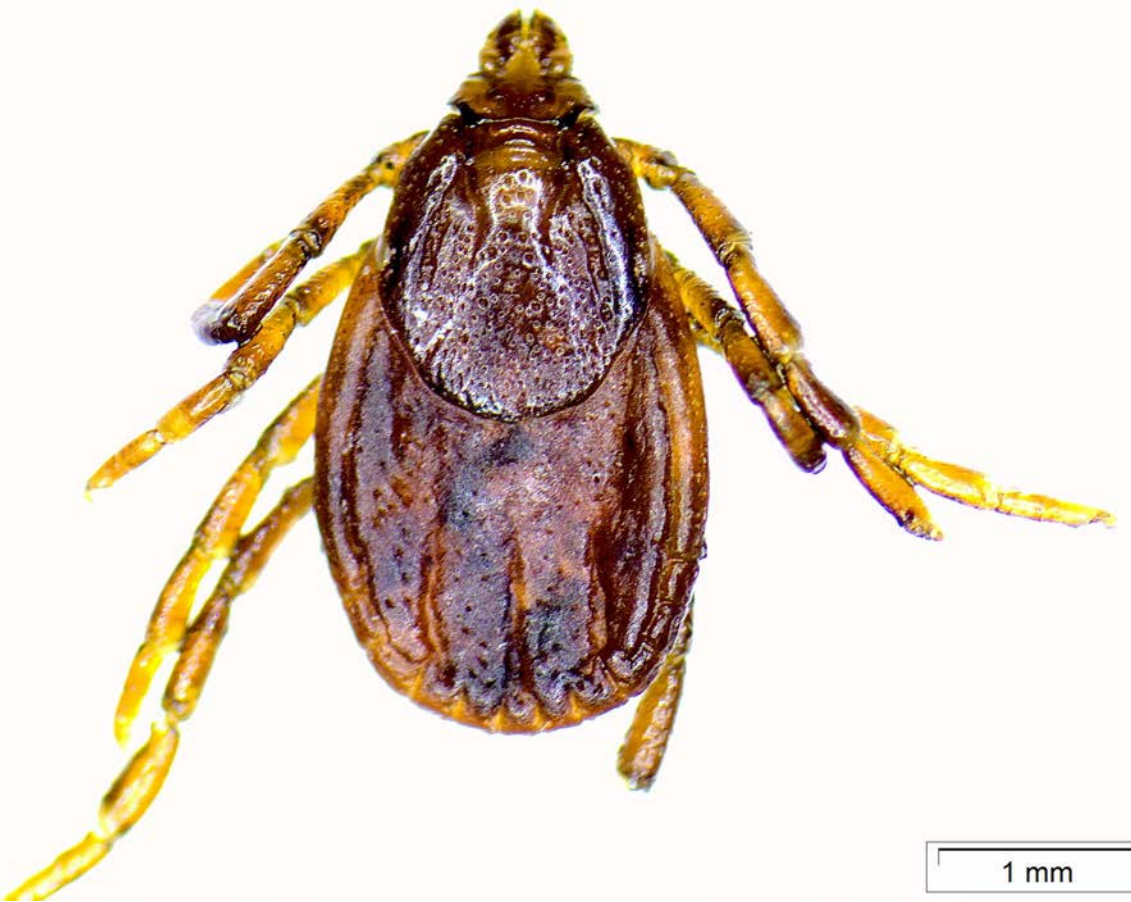

T

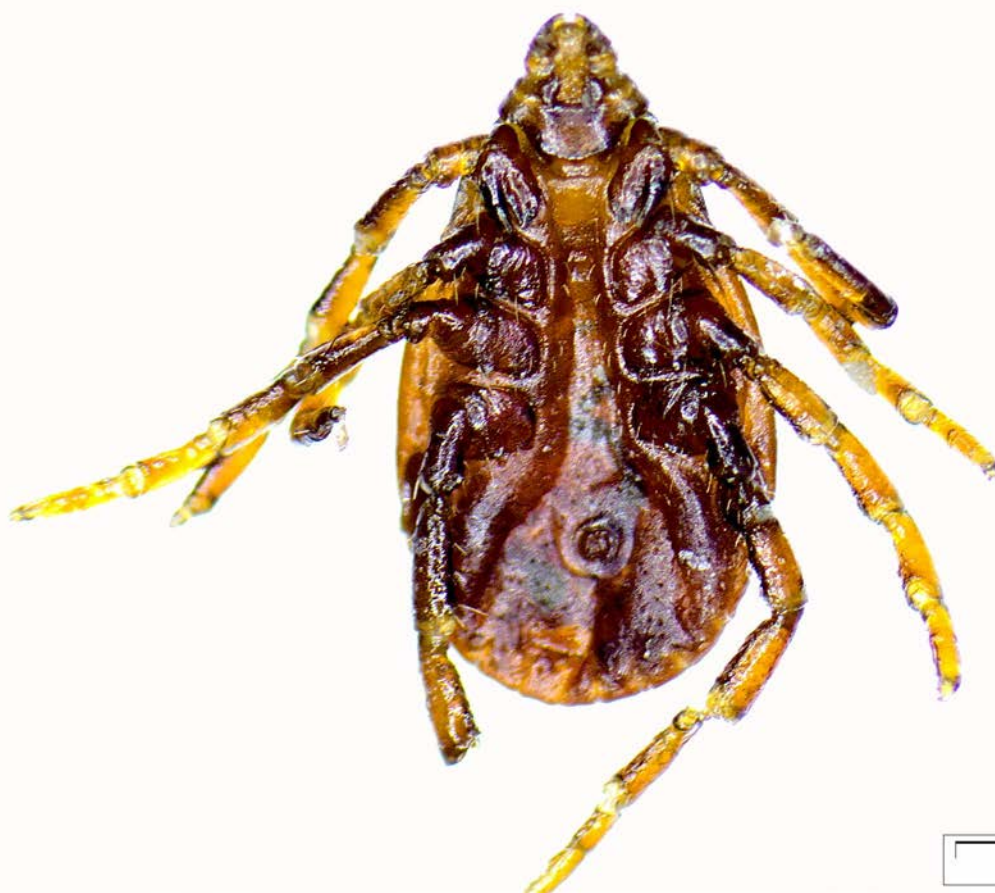

Supplement: Additional file 2: — Dorsal and ventral photographs of female ixodids. A) Dorsal view of Amblyomma triguttatum triguttatum. B) Ventral view of A. t. triguttatum. C) Dorsal view of Haemaphysalis bancrofti. D) Ventral view of H. bancrofti. E) Dorsal view of Haemaphysalis longicornis. F) Ventral view of H. longicornis. G) Dorsal view of Ixodes cornuatus. H) Ventral view of I. cornuatus. I) Dorsal view of Ixodes hirsti. J) Ventral view of I. hirsti. K) Dorsal view of Ixodes myrmecobii. L) Ventral view of I. myrmecobii. M) Dorsal view of Ixodes holocyclus. N) Ventral view of I. holocyclus. O) Dorsal view of Ixodes tasmani. P) Ventral view of I. tasmani. Q) Dorsal view of Rhipicephalus australis. R) Ventral view of R. australis. S) Dorsal view of Rhipicephalus sanguineus. T) Ventral view of R. sanguineus. Individual tick specimens were collected from the following localities: the township of Gidgegannup, WA (A-B); the town of Missabotti, NSW (C-D); the town of Bellingen, NSW (E-F); the city of Devonport, TAS (G-J, O-P); the town of Esperance, WA (K-L); the town of Byangum, NSW (M-N); the town of Sarina, QLD (Q-R); the Indigenous Australian community of Mutitjulu, NT (S-T). (PDF 1501 kb) [file 13071_2016_1480_MOESM2_ESM.pdf]
