# Supplementary material for: A survey of ticks (Acari: Ixodidae) of companion animals in Australia
Source: Parasit Vectors. 2016 May 10;9:207. doi: 10.1186/s13071-016-1480-y (PMC4862205; doi:10.1186/s13071-016-1480-y)
Supplement: Additional file 3: — Collection localities and number of ticks (Acari: Ixodidae) recorded on dogs (Canis lupus familiaris), cats (Felis catus), and horses (Equus ferus caballus). (PDF 241 kb) [file 13071_2016_1480_MOESM3_ESM.pdf]

Additional file 3

Collection localities and number of ticks (Acari: Ixodidae) recorded on dogs (*Canis lupus familiaris*), cats (*Felis catus*), and horses (*Equus ferus caballus*)

**Host species: *Canis lupus familiaris***						
**State**	**Tick species**	**Localities**	**Number of hosts**	**Number of instars**	**Latitude**	**Longitude**
New South Wales	*Haemaphysalis bancrofti*	Missabotti	1	1 female	-30.577003	152.792051
		North Bega	2	2 nymphs<br>1 female	-36.66665	149.824444
	Haemaphysalis longicornis	Bellingen	1	1 female	-30.453977	152.900557
		Missabotti	5	26 nymphs<br>9 females	-30.576993	152.792308
			4	3 nymphs<br>3 females	-30.565893	152.762577
			17	87 nymphs<br>37 females	-30.577003	152.792051
		Sydney	1	1 female	-33.870000	151.210000
		Thumb Creek	1	9 nymphs	-30.683660	152.620824
		Verona	6	2 nymphs<br>10 females	-36.445374	149.839611
	Ixodes holocyclus	Bellingen	5	6 females	-30.453977	152.900557
		Bowraville	1	1 female	-30.641113	152.710640
			1	1 female	-30.633323	152.804900
		Byron Bay	22	2 males<br>25 females	-28.636596	153.578101
		Callala Bay	1	1 female	-34.996702	150.721342
		Charlestown	6	7 females	-32.961852	151.697937
		Coffs Harbour	2	2 females	-30.305864	153.132592
		Culburra Beach	1	1 female	-34.933159	150.766710
			1	1 female	-34.933002	150.765954
		Greenwich	1	1 female	-33.832000	151.186000
		Hawkesbury River	1	1 female	-33.585000	151.215000
		Lane Cove	1	1 female	-33.818000	151.162000
		Lindfield	3	4 females	-33.777013	151.148352
			1	1 female	-33.776000	151.169000
		Marsfield	1	1 female	-33.784000	151.094000
		Merimbula	5	6 females	-36.888455	149.904951
			1	1 female	-32.154628	116.016502
		Missabotti	17	53 males<br>102 females	-30.577003	152.792051
			6	32 nymphs<br>4 males<br>51 females	-30.565893	152.762577
			5	17 males<br>27 females	-30.576993	152.792308
		Mona Vale	17	17 females	-33.675386	151.304081
		Moruya	13	1 male<br>17 females	-35.878388	150.088500
		Murrah	1	1 female	-36.513000	149.978000
		Narooma	4	5 females	-36.222237	150.127750
		North Bega	15	2 nymphs<br>1 male<br>24 females	-36.666650	149.824444
		Northbridge	1	1 female	-33.806890	151.223317
		Pambula	3	3 females	-36.932983	149.881750
		Peats Ridge	2	3 females	-33.372428	151.231090
		Roseville	1	1 female	-33.785000	151.177000

State	Tick species	Localities	Number of hosts	Number of instars	Latitude	Longitude
New South Wales	*Ixodes holocyclus*	Seaforth	19	22 females	-33.797860	151.250484
		Tanja	1	2 nymphs	-36.628000	149.933000
			2	2 females	-36.628000	149.933000
		Thumb Creek	1	1 female	-30.683660	152.620824
		Turramurra	15	1 nymph<br>17 females	-33.727743	151.141466
			5	2 nymphs<br>6 females	-33.727720	151.141477
		Wagga Wagga	1	1 female	-35.145553	147.373946
		Wollstonecraft	1	1 female	-33.828000	151.194000
		Wyneden	1	1 female	-28.527000	152.906000
		Wyoming	8	8 females	-33.411597	151.348814
	Ixodes tasmani	Seaforth	1	1 female	-33.797860	151.250484
	Rhipicephalus sanguineus	Medowie	3	6 females	-32.740776	151.863641
Northern Territory	*Rhipicephalus sanguineus*	Bagot	6	48 nymphs<br>74 males<br>58 females	-12.415000	130.856000
		Kulaluk Community	1	10 larvae<br>1 nymph<br>4 males<br>3 females	-12.398000	130.852000
		Katherine	5	1 larva<br>1 nymph<br>8 males<br>13 females	-14.461669	132.262805
			10	10 nymphs<br>65 males<br>57 females	-14.460338	132.265468
		Knuckey Lagoon	1	7 females	-12.426825	130.934141
		Alpurrurulam Community	3	2 nymphs<br>26 males<br>22 females	-20.981094	137.861604
		Minmarama Community	1	15 nymphs	-12.411181	130.849181
		Mutitjulu Community	3	57 nymphs<br>71 males<br>52 females	-25.352097	131.061208
			5	3 nymphs<br>111 males<br>135 females	-25.352300	131.066700
		Nyirripi Community	1	12 nymphs<br>43 males<br>12 females	-22.647568	130.549449
		Palmerston Indigenous Village	12	16 larvae<br>55 nymphs<br>287 males<br>327 females	-12.488097	131.011681
		Palmerston	9	3 larvae<br>2 nymphs<br>17 males<br>23 females	-12.481784	130.986319
		Parap	15	12 nymphs<br>163 males<br>127 females	-12.430728	130.843786
		Tennant Creek	1	7 males<br>7 females	-19.648306	134.186642
		Yuelamu Community	1	4 males<br>2 females	-22.257958	132.204607

State	Tick species	Localities	Number of hosts	Number of instars	Latitude	Longitude
Northern Territory	*Rhipicephalus sanguineus*	Yuendumu Community	2	1 larva<br>22 nymphs<br>47 males<br>13 females	-22.253296	131.795945
Queensland	*Haemaphysalis bancrofti*	Atherton	1	1 female	-17.260609	145.477149
	Haemaphysalis longicornis	Atherton	1	1 nymph	-17.260609	145.477149
		Brisbane	3	2 larva<br>22 nymphs	-27.648539	153.151835
	Ixodes holocyclus	Atherton	28	4 nymphs<br>1 male<br>31 females	-17.260609	145.477149
		Beechmont	1	1 male<br>2 females	-28.121883	153.183992
		Brinsmead	18	19 females	-16.897619	145.714192
		Brisbane	29	9 nymphs<br>1 male<br>33 females	-27.648539	153.151835
		Cairns	1	1 female	-16.805092	145.690346
		Cooroy	1	1 female	-26.458734	152.952405
		Image Flat	3	3 females	-26.603379	152.946080
		Kuranda	28	4 nymphs<br>1 male<br>30 females	-16.839165	145.615755
		Mackay	4	3 nymphs<br>5 females	-21.166279	149.145550
			7	7 females	-21.150380	149.169320
		Mansfield	8	8 females	-27.530819	153.098645
		Nambour	13	23 nymphs<br>15 females	-26.621000	152.952000
		Ninderry	1	1 nymph	-26.545000	152.991000
		Palmwoods	3	3 females	-26.685042	152.958990
		Park Ridge	9	12 females	-27.689544	153.034032
		Sarina	10	1 nymph<br>13 females	-21.419860	149.215904
		Trinity Beach	12	1 nymph<br>12 females	-16.805747	145.689772
		Tully	24	1 nymph<br>24 females	-17.931684	145.924253
		Yeppoon	5	7 females	-23.127991	150.744685
	Ixodes tasmani	Sarina	2	4 females	-21.419860	149.215904
	Rhipicephalus australis	Sarina	1	1 nymph	-21.419860	149.215904
	Rhipicephalus sanguineus	Atherton	4	2 nymphs<br>1 male<br>1 female	-17.260609	145.477149
		Kuranda	1	8 males<br>10 females	-16.839165	145.615755
		Mackay	1	1 female	-21.150380	149.169320
		Sarina	4	1 nymph<br>2 males<br>5 females	-21.419860	149.215904
		Trinity Beach	2	8 males<br>8 females	-16.805747	145.689772
		Tully	2	2 males<br>6 females	-17.931684	145.924253
		Yeppoon	1	9 nymphs<br>1 male<br>1 female	-23.127991	150.744685
South Australia	*Rhipicephalus sanguineus*	Coober Pedy	5	1 nymph<br>31 males<br>14 females	-28.897000	134.797000

State	Tick species	Localities	Number of hosts	Number of instars	Latitude	Longitude
South Australia	*Rhipicephalus sanguineus*	Oodnadatta	10	5 larvae<br>54 nymphs<br>200 males<br>105 females	-26.797000	134.596000
Tasmania	*Bothriocroton* sp.	Northdown	1	1 nymph	-41.177000	146.486000
	Ixodes cornuatus	Calder	1	1 female	-41.105000	145.611000
		Devonport	6	3 nymphs<br>5 females	-41.184934	146.354899
		Latrobe	1	1 nymph	-41.236000	146.411000
		Lower Wilmot	3	3 nymphs	-41.352000	146.229000
	Ixodes holocyclus	Devonport	2	2 females	-41.184934	146.354899
	Ixodes tasmani	Devonport	28	16 larvae<br>2 nymphs<br>30 females	-41.184934	146.354899
			1	1 female	-41.194099	146.333333
			1	1 female	-41.186157	146.360742
		East Devonport	1	1 female	-41.180000	146.370000
		Lower Wilmot	9	10 females	-41.352000	146.229000
		Merseylea	1	1 female	-41.358000	146.442000
		Northdown	1	2 nymphs<br>6 males<br>6 females	-41.177000	146.486000
		Port Sorell	2	2 females	-41.166000	146.550000
		Sassafras	5	5 females	-41.256000	146.534000
Victoria	*Bothriocroton* sp.	Caldermeade	1	1 female	-38.246926	145.568947
		Dandenong	4	11 larvae	-37.847282	145.361500
		Willow Grove	1	1 male	-38.089560	146.200605
	Ixodes cornuatus	Mallacoota	2	2 nymphs	-37.546605	149.746596
	Ixodes tasmani	Dandenong	2	2 females	-37.847282	145.361500
Western Australia	*Amblyomma triguttatum triguttatum*	Bedfordale	1	1 female	-32.184961	116.059958
		Mount Helena	5	5 nymphs<br>2 females	-31.877632	116.210405
		Perth	1	1 female	-32.154628	116.016551
		Woodridge	1	1 female	-31.344637	115.599431
	Ixodes holocyclus	Tambellup	1	1 female	-33.973049	117.499575
	Ixodes myrmecobii	Esperance	3	3 females	-33.861000	121.891000
		Hopetoun Beach	1	1 female	-33.851000	120.156000
	Rhipicephalus sanguineus	Beaconsfield	1	1 female	-32.068000	115.764000
		Broome	1	2 males<br>5 females	-17.955000	122.241000
		Bull Creek	3	1 male<br>2 females	-32.054550	115.866458
		Cable Beach	5	19 nymphs<br>4 males<br>3 females	-17.946000	122.209000
		Carnarvon	5	14 males<br>28 females	-24.871514	113.676197
		Halls Creek	2	3 nymphs<br>7 males<br>16 females	-18.224000	127.667000
		Kalumburu Community	5	125 larvae<br>110 nymphs<br>8 males<br>6 females	-14.287000	126.634000
		Karratha	2	35 nymphs<br>1 female	-20.737000	116.846000
		Kurnangki Community	4	23 males<br>21 females	-18.194272	125.568678
		Marble Bar	1	1 male<br>2 females	-20.990000	119.626000

State	Tick species	Localities	Number of hosts	Number of instars	Latitude	Longitude
		Mindi Rardi Community	4	7 larvae<br>10 males<br>9 females	-18.194272	125.568678
Western Australia	*Rhipicephalus sanguineus*	Murdoch	3	1 nymph<br>42 males<br>3 females	-32.068626	115.833016
		Perth	4	4 females	-32.154628	116.016551
			5	3 males<br>9 females	-32.119016	115.955365
		Pinjar	1	6 females	-31.682000	115.866000
**Host species: *Felis catus***						
**State**	**Tick species**	**Localities**	**Number of hosts**	**Number of instars**	**Latitude**	**Longitude**
New South Wales	*Haemaphysalis bancrofti*	Eungai Creek	1	1 female	-30.802878	152.887626
	Ixodes holocyclus	Bellingen	3	4 females	-30.453977	152.900557
		Byangum	1	2 females	-28.361000	153.385000
		Byron Bay	5	1 male<br>5 females	-28.636596	153.578101
		Callala Bay	1	1 female	-34.993563	150.718269
		Charlestown	2	2 females	-32.961852	151.697937
		Coffs Harbour	1	2 females	-30.305864	153.132592
		Culburra Beach	1	2 females	-34.936020	150.772844
		Eungai Creek	2	1 nymph<br>4 males<br>5 females	-30.802878	152.887626
		Gulmarrad	5	4 nymphs<br>1 male<br>1 female	-29.499026	153.238612
		Kempsey	1	1 female	-31.079863	152.833976
		Killarney Heights	1	4 nymphs<br>1 male<br>2 females	-33.771409	151.222570
		Lindfield	1	4 females	-33.777013	151.148352
		Merimbula	2	2 females	-36.888455	149.904951
		Missabotti	1	4 females	-30.576993	152.792308
		Mona Vale	7	8 females	-33.675386	151.304081
		Moruya	9	13 female	-35.910650	150.085324
		Narooma	1	1 female	-36.222237	150.127750
		North Bega	2	3 females	-36.666650	149.824444
		Seaforth	8	14 females	-33.797860	151.250484
		St Ives	1	1 female	-33.731000	151.159000
		Stanmore	1	1 female	-33.887163	151.169007
		Turramurra	8	1 nymph<br>9 females	-33.727743	151.141466
			3	1 nymph<br>1 male<br>1 female	-33.733000	151.130000
		Wollongbar	10	11 nymphs<br>1 male	-28.816695	153.394647
	Rhipicephalus sanguineus	Medowie	1	1 female	-32.740776	151.863641
Queensland	*Ixodes holocyclus*	Atherton	12	12 females	-17.260609	145.477149
		Brinsmead	7	1 nymph<br>7 females	-16.897619	145.714192
		Brisbane	1	2 females	-27.648539	153.151835
		Kuranda	7	7 females	-16.839165	145.615755
		Mackay	2	2 females	-21.166279	149.145550
			1	1 female	-21.150380	149.169320
		Palmwoods	2	2 females	-26.685042	152.958990
		Park Ridge	4	6 females	-27.689544	153.034032
		Sarina	4	5 females	-21.419860	149.215904

State	Tick species	Localities	Number of hosts	Number of instars	Latitude	Longitude
Queensland	*Ixodes holocyclus*	Trinity Beach	3	1 nymph<br>3 females	-16.805747	145.689772
		Tully	16	17 females	-17.931684	145.924253
Tasmania	*Ixodes cornuatus*	Devonport	1	1 female	-41.184934	146.354899
	Ixodes hirsti	Devonport	1	1 female	-41.184934	146.354899
	Ixodes tasmani	Blackstone Heights	1	1 female	-41.466690	147.080704
Tasmania	*Ixodes tasmani*	Devonport	3	21 larvae<br>1 nymph<br>1 female	-41.184934	146.354899
			1	2 larvae<br>4 nymphs	-41.176000	146.351000
		East Devonport	1	1 female	-41.180000	146.370000
		Launceston	1	1 nymph	-41.445000	147.139000
		Lower Wilmot	1	1 nymph	-41.352000	146.229000
		Port Sorell	2	4 females	-41.166000	146.550000
		Sassafras	1	1 nymph	-41.256000	146.534000
Victoria	*Ixodes tasmani*	Trafalgar	1	1 nymph	-38.209322	146.154687
Western Australia	*Ixodes myrmecobii*	Esperance	1	1 female	-33.861000	121.891000
**Host species: *Equus ferus caballus***						
**State**	**Tick species**	**Localities**	**Number of hosts**	**Number of instars**	**Latitude**	**Longitude**
New South Wales	*Amblyomma triguttatum triguttatum*	Rowan Wood	1	1 female	-28.903000	150.723000
	Haemaphysalis bancrofti	Eungai Creek	2	1 nymph<br>2 males<br>3 females	-30.802878	152.887626
		Tanja	1	7 females	-36.644436	149.966990
	Haemaphysalis longicornis	Bellingen	3	23 nymphs<br>95 females	-30.428656	152.907869
		Bowraville	5	35 females	-30.641113	152.710640
		Byabarra	1	23 females	-31.532535	152.544901
		Eungai Creek	2	4 nymphs<br>7 females	-30.802878	152.887626
		Sancrox	1	14 females	-30.994187	150.376303
		Tanja	1	1 female	-36.628000	149.933000
		Wyrallah	1	1 female	-28.888000	153.300000
	Ixodes holocyclus	Bellingen	1	1 female	-30.453977	152.900557
		Bowraville	4	4 females	-30.641113	152.710640
		Eungai Creek	2	1 male<br>8 females	-30.802878	152.887626
		Tanja	1	1 female	-36.644436	149.966990
			1	5 nymphs<br>1 female	-36.628000	149.933000
		Wyrallah	2	1 male<br>18 females	-28.888000	153.300000
Queensland	*Amblyomma triguttatum triguttatum*	Yelarsa	1	4 females	-28.572000	150.753000
	Haemaphysalis bancrofti	Atherton	1	1 female	-17.260609	145.477149
		Currumbin Valley	4	2 nymphs<br>1 male<br>5 females	-28.210679	153.405361
	Haemaphysalis longicornis	Currumbin Valley	2	2 nymphs<br>2 females	-28.210679	153.405361
	Ixodes holocyclus	Atherton	2	3 males<br>21 females	-17.260609	145.477149
		Brinsmead	1	1 female	-16.897619	145.714192

**State**	**Tick species**	**Localities**	**Number of hosts**	**Number of instars**	**Latitude**	**Longitude**
Queensland	*Ixodes holocyclus*	Currumbin Valley	4	3 males<br>26 females	-28.210679	153.405361
		Kuranda	3	3 females	-16.839165	145.615755
	Ixodes tasmani	Kuranda	1	1 female	-16.839165	145.615755
	Rhipicephalus australis	Kuranda	1	1 nymph<br>2 females	-16.839165	145.615755
Western Australia	*Amblyomma triguttatum triguttatum*	Bullsbrook	1	1 female	-31.648081	116.047406
		Gidgegannup	7	1 nymph<br>6 females	-31.772573	116.137715
			1	1 female	-31.793000	116.197000
